# Natural Loss of *eyeless/Pax6* Expression in Eyes of *Bicyclus anynana* Adult Butterflies Likely Leads to Exponential Decrease of Eye Fluorescence in Transgenics

**DOI:** 10.1371/journal.pone.0132882

**Published:** 2015-07-14

**Authors:** Mainak Das Gupta, Sam Kok Sim Chan, Antónia Monteiro

**Affiliations:** 1 Biological Sciences, National University of Singapore, Singapore; 2 Biological Sciences, Universiti Tunku Abdul Rahman, Kampar, Perak, Malaysia; 3 Yale-NUS College, Singapore; Alexander Fleming Biomedical Sciences Research Center, GREECE

## Abstract

Commonly used visible markers for transgenesis use fluorescent proteins expressed at the surface of the body, such as in eyes. One commonly used marker is the *3xP3-EGFP* cassette containing synthetic binding sites for the *eyeless/Pax6* conserved transcription factor. This marker cassette leads to fluorescent eyes in a variety of animals tested so far. Here we show that upon reaching adulthood, transgenic *Bicyclus anynana* butterflies containing this marker cassette exponentially loose fluorescence in their eyes. After 12 days, transgenic individuals are no longer distinguishable from wild type individuals. The decreased eye fluorescence is likely due to significantly decreased or halted *eyeless/Pax6* expression observed in wild type animals upon adult emergence. Implications from these findings include care in screening transgenic animals before these reach adulthood, or shortly thereafter, and in using adult animals of the same age for quantitative screening of likely homozygote and heterozygote individuals.

## Introduction

Transgenesis is a powerful technique to study the function or the regulatory information contained in DNA. Commonly, genomic insertions of test candidate DNA sequences are accompanied by the insertion of a marker gene that helps in the identification of transgenic individuals. Some of the most widely used markers of transgenesis are fluorescent proteins, such as Enhanced Green Fluorescent Protein (EGFP), Ds Red and others. These are inert proteins that are clearly visualized upon light excitation in live organisms once expressed, and allow the clear identification of a transgenic organism [1–3].

For fluorescent proteins to be expressed they need to be driven by an enhancer or a promoter sequence. Arguably, an ideal regulatory sequence should lead to fluorescent marker gene expression early during development, at the surface of the body, in a small region of the body, and continuously throughout development and adulthood. These marker expression characteristics allow for 1) early discard of non-transgenic organisms, saving time and resources in the rearing and screening process; 2) easy visibility and flexibility in the exact moment of screening and later maintenance of the line; and they 3) minimize conflict of fluorescent signals if tissues from the transgenic line are later going to be targeted with other fluorescent markers, such as in experiments involving antibody stainings using secondary antibodies labeled with fluorescent proteins.

One of the most common markers for transgenesis is a “cassette” consisting of a synthetic *3xP3* promoter driving *EGFP* [4]. This promoter contains three binding sites for the conserved *eyeless* (*Pax6*) transcription factor, and drives gene expression in the eyes and central nervous of many animals tested so far. These include flies, mosquitoes, butterflies, moths, beetles, crustaceans, and planarians [4–10]. In addition, this marker cassette meets most of the requirements for an ideal transgenic marker mentioned above. Eyes and the central nervous system are differentiated early during embryogenesis, hence allowing for early screening; eyes, in particular, differentiate on the surface of the body, and grow over the course of development, allowing for high visibility of the marker and flexibility in moment of screening; and eyes and central nervous tissue make up a small portion of the overall cells/tissue in a body, allowing most tissue in the body to be non-fluorescent and subject to further characterization with fluorescent probes.

Recently, however, we noticed that a transgenic line of *Bicyclus anynana* butterflies, containing a *3xP3-EGFP*-marker cassette [11, 12], was showing variable patterns of EGFP fluorescence in adult eyes. Some adult eyes, in fact, appeared almost as dim as wild type eyes. Here we investigate the cause of such adult eye brightness variability.

Several studies have assumed that *eyeless* (*Pax6*) is required to drive the expression of genes containing the P3 binding element during the later stages of compound eye development in *Drosophila melanogaster* [13, 14]. However, the precise function of *eyeless* during these stages of eye development has not been established. Furthermore, recently it was also shown that the combined function of two transcription factors in flies, namely *PvuII-PstI homology 13* (*Pph13)* and *orthodenticle*, was required for proper development of eyes in the later stages [14]. Importantly, *Pph13* was shown to be necessary and sufficient to drive the expression of a reporter placed under the *3xP3* promoter. It was also shown that *Pph13* and *eyeless* recognize the same consensus binding sequence [14]. Therefore, we hypothesized that the reduction in *EGFP* expression in adult eyes in *B*. *anynana* could be explained by the reduction in the expression of either *eyeless*, *Pph13*, or both of these regulatory genes during late pupal stages and/or early adults of this species.

Below we detail a series of experiments that test these hypotheses. We then discuss the implications of our results regarding the identified shortcoming of the *3xP3-EGFP* marker cassette, and propose ways to overcome it.

## Materials and Methods

### Animal husbandry

*B*. *anynana* were reared at 26°C, and 60–80% humidity. Larvae were fed on young corn plants. Adults were fed on moist banana slices. Animals were monitored for day of emergence, day 1, and given a unique number on their wings with a sharpie marker. Wild type as well as transgenic *B*. *anynana* butterflies containing the *3xP3-EGFP* marker cassette were used for comparisons of eye brightness across time. Transgenic butterflies belonged to a *Ultrabithorax* over-expression line which also expresses *EGFP* under control of the *eyeless* gene via the *3xP3* promoter. The making and characterization of this line is described elsewhere [11, 12].

### Eye brightness analysis

Transgenic and wild-type butterflies were repeatedly imaged for eye brightness over the course of their lives in order to detect changes in eye brightness levels with age. Butterflies were held by their wings with flexible forceps, and the head was placed under a stereo fluorescent microscope (Leica M165 FC) in a lateral position. Right eyes of adult animals were imaged and photographed under blue light illumination using a GFP filter set (excitation filter ET470/40 nm—emission filter ET525/50 nm). Frequency of imaging of the same animal varied between 2 and 3 days. On a single day, left eyes from all animals of different ages were also photographed. Digital images of eyes were analyzed for brightness levels using Adobe Photoshop. First the eye was delineated with the lasso tool, then the pixels inside the lasso tool were averaged, the image was converted to Grey Scale, and the K-values (darkness) of the pixels were obtained using the color picker tool (K value range: 0–100). We obtained brightness levels by subtracting K from 100.

### Statistical analysis

Differences in brightness levels across left and right eyes of individuals of different ages were tested using a paired-samples T-test using IBM SPSS Statistics, version 21.

Graphs were plotted in Microsoft Excel for Mac 2011, version 14.4.4.

### RNA extraction from heads and semi-quantitative RT-PCR

Optic lobes were separated from brains, and total RNA was extracted separately from these two brain regions from pupae and adults of different ages using TRIzol Reagent (Life Technologies). Two sets of animals (with the same exact ages) were used for these experiments. In brief, tissue was homogenized in 500 μl TRIzol using metal beads, phase separated using 100 μl chloroform followed by precipitation of RNA from the aqueous phase using an equal volume of iso-propanol. Precipitated RNA was rinsed with 75% ethanol, dried, dissolved in RNase free water and stored at -20°C. The RNA was treated with DNase I (Thermo Scientific) to remove any genomic DNA contamination before proceeding with cDNA synthesis. First strand cDNA was synthesized from 2μg of total RNA and random primers using the RevertAid First Strand cDNA Synthesis Kit (Thermo Scientific) following manufacturer's recommendations.

A full length *B*. *anynana eyeless* sequence was identified from an adult head transcriptome dataset (a few hours upon emergence; Macia Munoz, unpublished) (S1 File). Forward and reverse primers flanking the paired domain and homeodomain (Table 1) were used to study *eyeless* expression in wild type individuals (S1 File). PCR was run for *EGFP* in transgenic individuals, as well as *eyeless* in wild type individuals, and the housekeeping gene *Elongation factor-1 alpha* (*EF1a*) in both sets of individuals, using these and other primer sets (Table 1). PCR was performed using Taq DNA Polymerase (NEB) following manufacturer's recommendations. An annealing temperature of 57°C was used in all cases. The number of PCR cycles required for keeping the amplification in the exponential phase was determined empirically for each gene separately. A total of 29 amplification cycles were performed for all reactions.

**Table 1:** Primers used in this study.

Primer Name	Sequence (5’ to 3’)
EGFP– 373—forward	CGTCCAGGAGCGCACCATCTTCTT
EGFP– 373—reverse	ATCGCGCTTCTCGTTGGGGTCTTT
Paired domain—I_F	GGAACCCACCACGGCGGC
Paired domain—I_R	CCTTCGCTAAGTAGGCGGTCTC
Paired domain—II_F	CTGACTACAAGCGGGAGTG
Paired domain—II_R	GGTAGTGGTGCTGGTAGCG
Homeodomain—III_F	GCGAACGCACTACCCGG
Homeodomain—III_R	CGCCAGACATTGAGCTATACG
EF1a—forward	GTGGGCGTCAACAAAATGGA
EF1a—reverse	TTAGCGGCAAAAACAACGAT

## Results

### GFP fluorescence decreases exponentially in the adult flies post emergence

**Fig 1:**
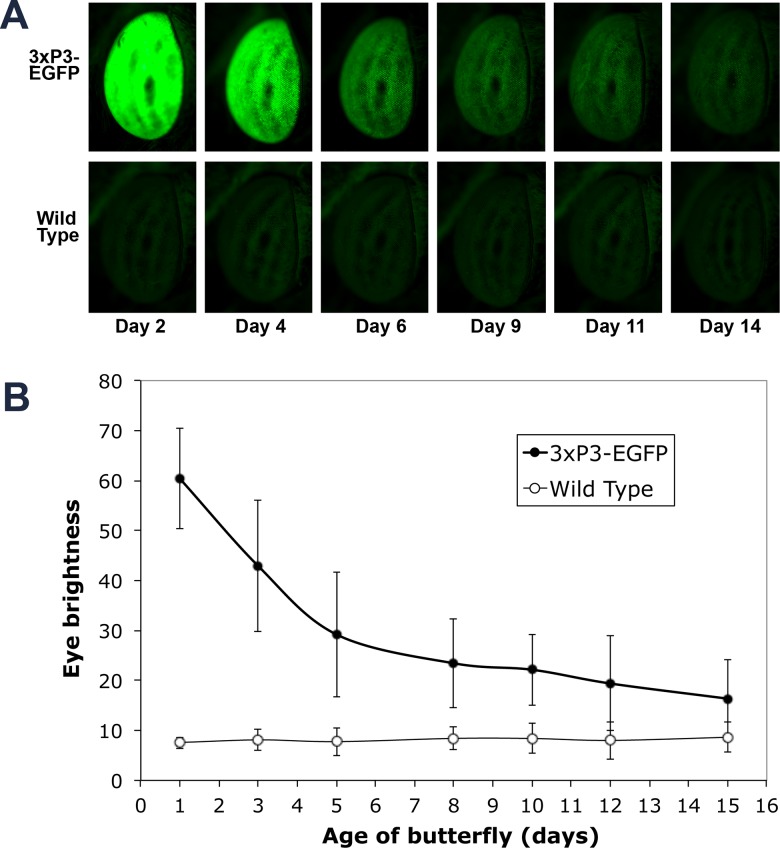
Eye brightness of transgenic (3xP3-EGFP) butterflies decreases over the course of adulthood. A) Brightness of right eyes of the same representative transgenic and wild type individuals over the course of 14 days. B) Quantitative variation in mean eye brightness over the course of 15 days for 3xP3-EGFP (n = 45) and Wt (n = 22) individuals. Error bars represent 1 SD.

We observed that fluorescent levels in transgenic adult individuals decreased with time in an exponential fashion (Fig 1A). Eye brightness of wild-type individuals, however, remained constant and low throughout. By day 12 after adult emergence, the brightness levels of transgenic and wild-type animals were overlapping (Fig 1B), indicating that reliable identification of a transgenic animal of this age was no longer possible. Imaging the eyes multiple times did not have a significant effect on eye brightness levels. Left eyes, imaged once, had comparable brightness levels to right eyes, imaged a variable number of times (t = 0.93, p = 0.366, df = 16). The correlation between the two measurements was also high and significant (r = 0.879, p<0.001, n = 17).

### Reduction in GFP fluorescence correlates with reduced expression of *eyeless* in adults

**Fig 2:**
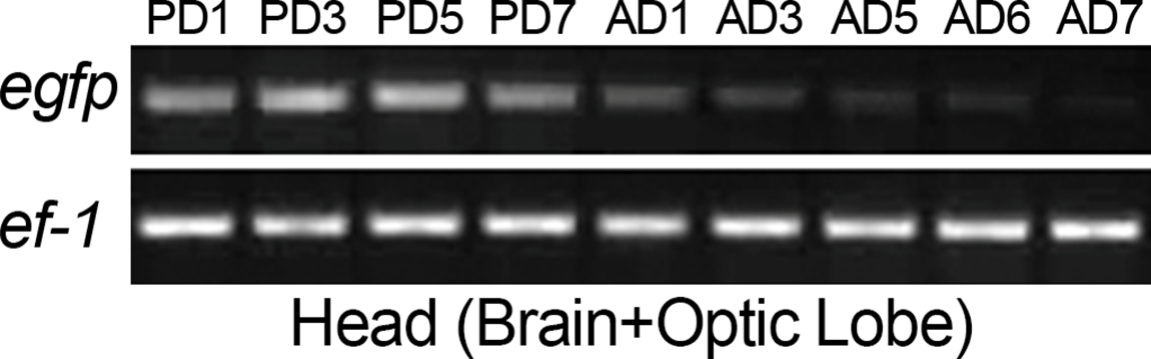
Expression of *EGFP* decreases upon adult emergence. Levels of *EGFP* and the house-keeping gene *Elongation Factor 1-alpha* cDNA, present in head samples of transgenic pupae and adults of different ages inferred with semi-quantitative RT-PCR. PD- Pupal Day and AD- Adult Day.

To test whether the reduced levels of eye fluorescence in the transgenic line were due to reduced expression of *EGFP*, we extracted mRNA from heads of transgenic pupae and adults of different ages and performed RT-PCR on these samples. *EGFP* was strongly expressed in the head during all the pupal stages while the expression started decreasing post emergence and was markedly reduced by day 5 of adulthood (Fig 2). After observing the decline of EGFP expression in the transgenic adults we tested whether this decline could be explained by the decrease in the expression of regulatory genes *eyeless* or *Pph13* in wild type eye development.

**Fig 3:**
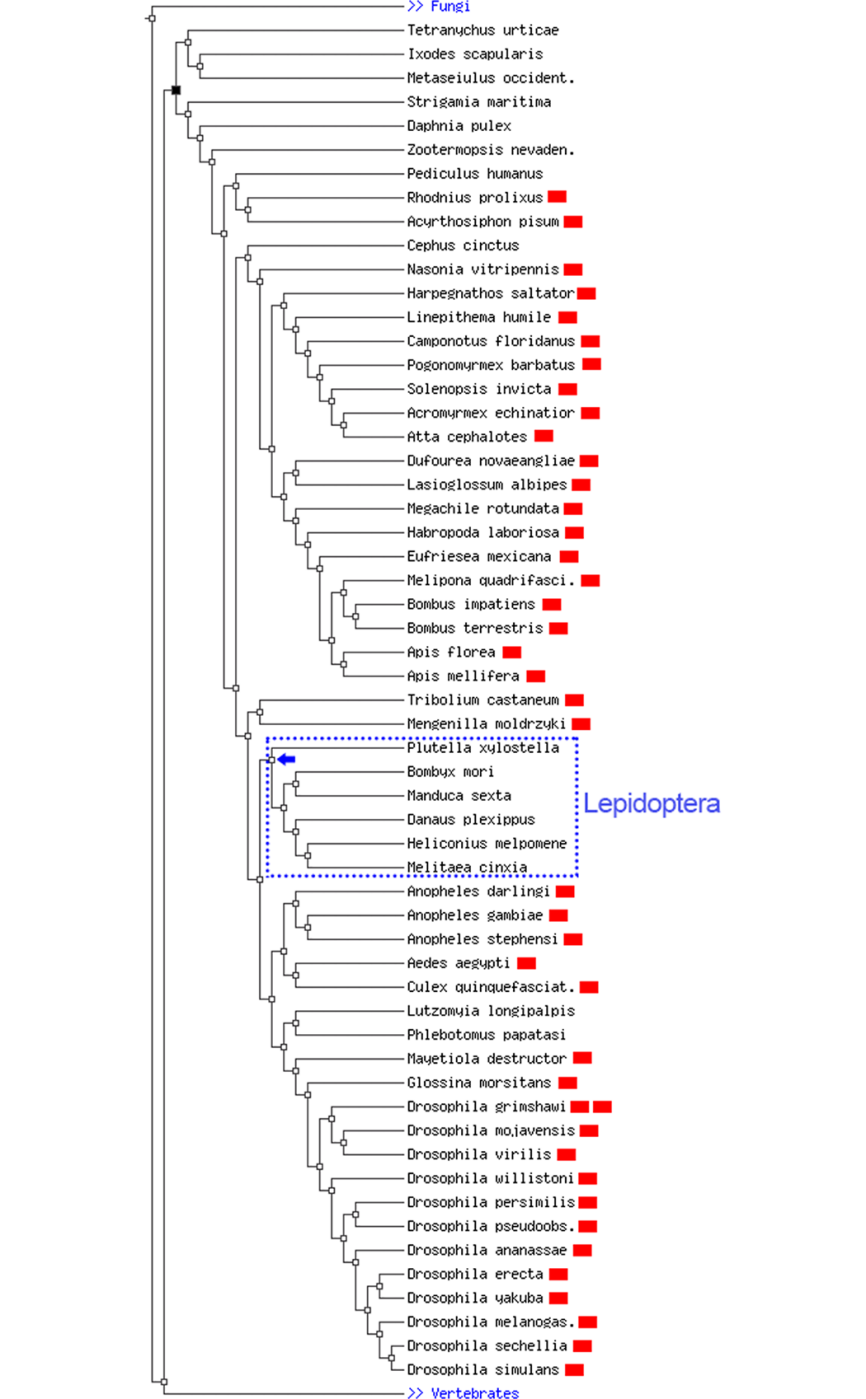
Phylogenetic tree showing the relationship among 57 arthropod species and the presence or absence of the *Pph13* orthologs. Out of the 57 species, 41 showed the presence of at least one copy of Pph13 in their genomes (indicated by red rectangle). A clear ortholog of Pph13 was specifically missing in the lepidopteran clade. Blue arrow shows the point of probable loss of this gene in this clade. The phylogenetic tree was adapted from OrthoDB (http://cegg.unige.ch/orthodb7).

An analysis of orthologous genes across 57 species of arthropods using *D*. *melanogaster Pph13* gene as the query in OrthoDB (http://cegg.unige.ch/orthodb7) [15] showed that 41 out of the 57 species have at least one copy of this gene. Interestingly, an ortholog of this gene was specifically missing in the lepidopteran clade (Fig 3). Additionally, searching the genome databases of *B*. *anynana* (www.bicyclus.org) and other lepidopterans like *Danaus plexippus* (MonarchBase, http://monarchbase.umassmed.edu/home.html), *Bombyx mori* (SilkDB, http://www.silkdb.org/silkdb/) and other butterflies (Heliconius Genome Project, http://www.butterflygenome.org/), using the same query sequence, did not yield any significant matches outside the conserved homeodomain. On the other hand, the orthologs of *eyeless* and *orthodenticle* were present in all the 57 arthropod species listed in OrthoDB (not shown). Therefore, we reasoned that the reduction in *EGFP* levels in the adult butterfly eyes could be the result of reduced *eyeless* expression. Since *eyeless* is known to be expressed in the optic lobes as well as the central nervous system in flies [13, 14], we separated the brain from the optic lobes of pupae and adult wild type butterflies before extracting the total RNA from these structures.

**Fig 4:**
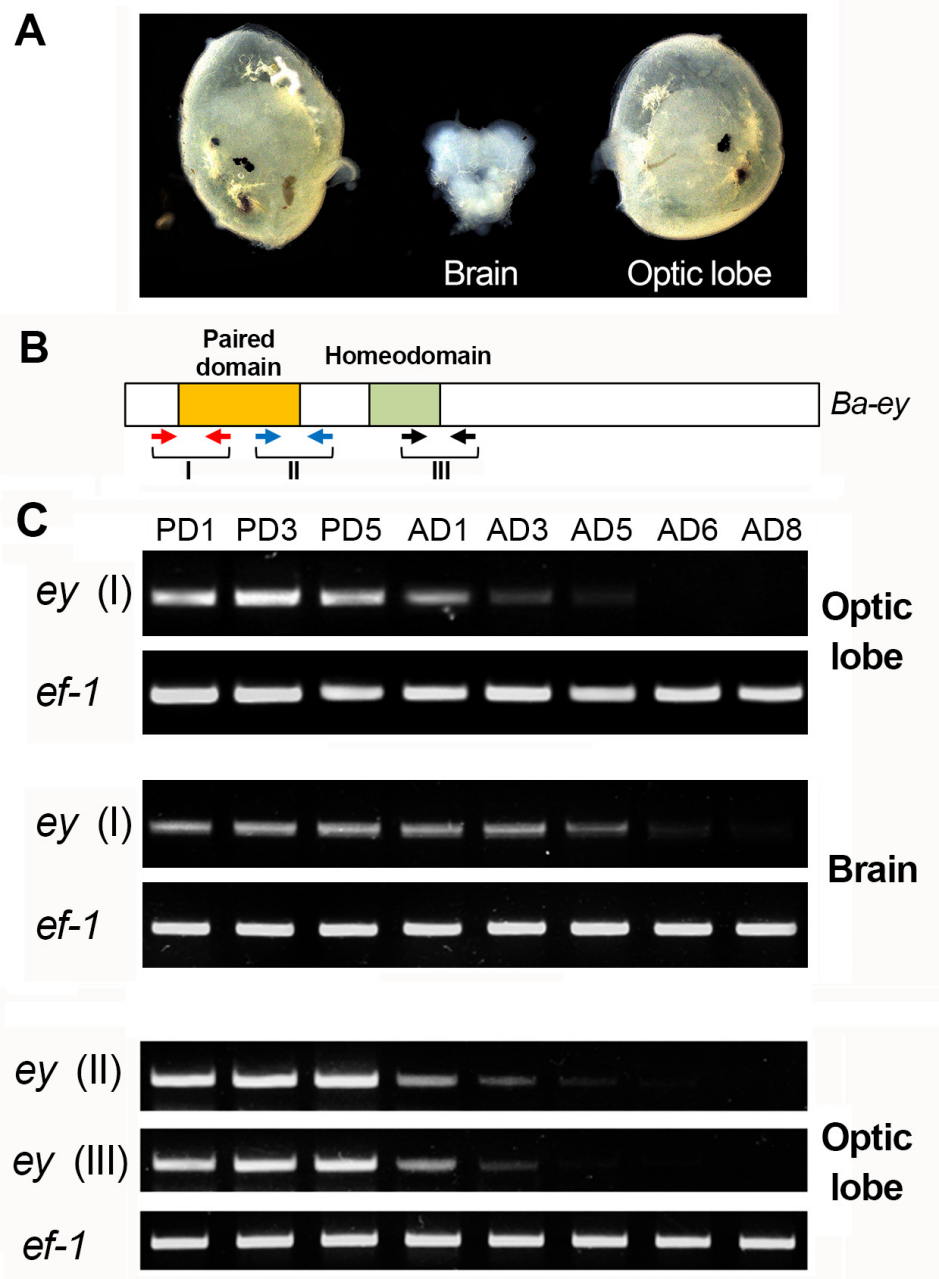
Expression of *eyeless* decreases upon adult emergence. (A) Dissected brain and optic lobes of a 2-day old pupa of *B*. *anynana*. (B) Schematic of the *B*. *anynana eyeless* gene and the size and position of the three amplicons (I, II, and III), and respective primers, used to monitor *eyeless* expression levels. (C) Expression levels of the three *eyeless* amplicons in the optic lobes and brain of pupae and adult butterflies of different ages inferred with semi-quantitative RT-PCR. *Elongation Factor 1-alpha* (*ef-1*) was used as the internal control. PD- Pupal Day and AD- Adult Day.

At least two different isoforms of *eyeless* are expressed in the developing eye discs of *Drosophila* due to alternative splicing [16]. The protein isoforms differ in length by 60 amino acids and bind to the same target sites with different affinities. It has been suggested that an equilibrium in the levels of these two isoforms may be necessary for proper eye development [16]. Both isoforms contain the conserved DNA-binding homeodomain and the paired-domain. Since the latter is necessary for binding to the *3xP3* promoter [13, 14], we designed primers around the paired domain of the gene to study the expression levels of *eyeless* in *B*. *anynana* optic lobes (Fig 4B). In addition, we also investigated *eyeless* expression using primers that amplify the homeodomain (Fig 4B).

Semi-quantitative RT-PCR showed strong expression of *eyeless* in the optic lobes as well as the brain of wild type animals during the pupal stages but showed a significant reduction in adult optic lobes starting on day 1 post emergence and in brains starting a little later (Fig 4C). This result was observed with primer pairs flanking both the paired domain and homeodomain (Fig 4C). By the 5^th^ day post emergence, *eyeless* expression was almost undetectable in the optic lobes. Since the expression pattern of *EGFP* closely matches that of *eyeless* in the optic lobes, we propose that the reduction of fluorescence in the adult eyes of *B*. *anynana* is due to the reduction in *eyeless* expression upon emergence of the butterflies.

## Discussion

Here we discover that the *3xP3-EGFP* marker cassette, which has been widely used as a marker for transgenesis in a variety of organisms [4–10] has one shortcoming in butterflies: it does not effectively mark older transgenic animals. The transgenic line tested here had very high levels of eye fluorescence in freshly emerged adults but eye brightness became comparable to that of wild type eyes after 12 days. This reduction in fluorescence levels is likely due to the substantial reduction or arrest in *eyeless* (*Pax6*) gene expression that is known to drive the *3xP3* synthetic promoter. This likely led to low *EGFP* mRNA production, and likely low EGFP protein levels. The half-life of (E)GFP proteins in mammalian cell culture is around 26 hours [17]. This explains the exponential decay of fluorescence starting at day 1. These findings, while specific for the study organism described here, may be more generally applicable to other animals.

The 12-day “grace” period identified in this study may also be specific to this line of transgenic butterflies. Previous transgenic lines for *B*. *anynana* had substantially lower starting levels of eye fluorescence relative to the line examined here [7, 18, 19]. Variation in fluorescent levels across transgenic lines is commonplace and often attributed to regulatory sequences flanking the exact genomic insertion site of the marker cassette. Two new transgenic lines recently established in the lab have an even shorter grace period for screening. Larval and pupal eyes are extremely bright, but green fluorescence becomes extremely reduced by the last day of pupation and transgenic and wild-type animals look alike upon emergence.

*eyeless* is a conserved transcription factor involved in eye development across metazoans [20]. It is still unclear whether this gene has a function in the maintenance of gene expression in adult eyes, once development is complete. Our results suggest that strong *eyeless* expression is maintained in the optic lobes throughout the pupal stages of wild type animals and the reduction in expression starts in recently emerged adults (day 1; Fig 4C). No detectable levels of *eyeless* mRNA were present in day 6 wild type adults (Fig 4C). The pupal stages coincide with the differentiation of adult compound eyes and the loss of smaller simpler larval eyes, the stemmata. Overall, our data indicate that *eyeless* may be important for eye development but not eye maintenance in adult butterflies.

In *D*. *melanogaster*, the development of the adult eye structures seem to be regulated by a combined activity of *Pph13* and *otd* [21]. Since a clear ortholog of *Pph13* could not be detected in the sequenced lepidopteran species, it is possible that these species combine the activities of *eyeless* and *otd* in the generation of the adult eye structures.

Our finding highlights important precautions that should be taken when creating or maintaining transgenic lines that rely on the 3xP3 promoter as well as other promoters that rely on *eyeless/Pax6* to drive gene expression in the eyes. An example of the latter includes a lens crystalline enhancer driving the expression of fluorescent proteins in the eyes of transgenic mice [22, 23]. These precautions assume that, as in *B*. *anynana*, *eyeless/Pax6* is down regulated in eyes as the animals reach adulthood, but should obviously be verified in each individual system. The precautions are:

1. To not rely exclusively on screening transgenic lines during adulthood. If adulthood is the most convenient stage for screening, then ensure the animals are screened in the first few days after reaching adulthood.
2. To use adult individuals of exactly the same age to quantify fluorescent levels to distinguish homozygotes from heterozygotes. A few studies, including previous work from this lab, have relied on quantifying the intensity of eye fluorescence in adults to establish homozygous lines [11, 22]. We suggest that age be kept constant otherwise no reliable identification of genotype can be done.

## Supporting Information

S1 File *Bicyclus anynana eyeless* sequences and the primers used in the RT-PCR experiments. (A) *Bicyclus anynana eyeless* mRNA sequence. The regions coding for the paired domain and the homeodomain are highlighted in green and yellow, respectively. The positions of the primers are underlined. F and R refer to forward and reverse primers, respectively. (B) Sequence of amplicons using the primers used in the study. (C) Alignment of the sequenced regions to the *eyeless* mRNA highlighting the paired domain and homeodomain.
(DOCX)
