# Supplementary material for: Natural Loss of eyeless/Pax6 Expression in Eyes of Bicyclus anynana Adult Butterflies Likely Leads to Exponential Decrease of Eye Fluorescence in Transgenics
Source: PLoS One. 2015 Jul 14;10(7):e0132882. doi: 10.1371/journal.pone.0132882 (PMC4501736; doi:10.1371/journal.pone.0132882)
Supplement: S1 File — (A) Bicyclus anynana eyeless mRNA sequence. The regions coding for the paired domain and the homeodomain are highlighted in green and yellow, respectively. The positions of the primers are underlined. F and R refer to forward and reverse primers, respectively. (B) Sequence of amplicons using the primers used in the study. (C) Alignment of the sequenced regions to the eyeless mRNA highlighting the paired domain and homeodomain. (DOCX) [file pone.0132882.s001.docx]

**Supplementary File 1. *Bicyclus anynana eyeless* sequence and the primers used in the RT-PCR experiments**

(A) *Bicyclus anynana eyeless* mRNA sequence. The regions coding for the paired domain and the homeodomain are highlighted in green and yellow, respectively. The positions of the primers are underlined. F and R refer to forward and reverse primers, respectively.

**>Ey_Complete**

CCGCCACCACGCTGGTGGGGGGGCTGGTGGCGCCGCCGCGGGAACCCACCACGGCGGCAGAAGCCTTCTGGAAGATG

I-F

CCGCACAAAGATGAGCTGATGCACAGTGCGGCAATGGGTGGCGGCGCCCTCTTCGGGTGCTCCTCTGCCGGCCACAGCGGGATCAACCAGCTGGGCGGGGTGTACGTCAACGGGAGACCCTTGCCTGACTCCACGCGGCAGAAGATAGTGGAGCTGGCGCACTCGGGGGCGCGGCCGTGCGACATCAGCAGGATCCTGCAGGTGTCCAACGGCTGCGTGTCCAAGATACTA

I-R

GGCAGGTACTACGAGACGGGGTCAATCAAGCCCCGTGCGATCGGCGGGTCAAAGCCGCGGGTCGCAACGACCCCGGTGGTTTCGAAGATCGCTGACTACAAGCGGGAGTGCCCTTCAATCTTCGCGTGGGAGATCCGGGATCGCCTGCTTAGCG

II-F

AAAACGTCTGCAATAACGACAATATCCCTAGCGTGTCATCAATCAACCGCGTGCTAAGGAACCTAGCATCACAGAAGGAGCAAGCAGCATCAGCGCAGAACGACAGCGTGTATGAGAAGCTGAGGATGTTCAATGGACAGGCAGCCACAGGGTGGTGGTACCCTGGACTACCCGCCGCGCCTACCGCACCCGCGCTACCAGCACCACTACCCCCCCAGCTGAATAGGCCGA

II-R

CGTCCGAAGACCATAAACGAGATACCTTACAATCGGAGGCCGGGTCGGACGGGAACAGCGAGCACGCGTCATCAGGAGACGAGGACTCGCAGATGAGGCTGAGGCTGAAGAGGAAGCTGCAGAGGAACCGGACCTCCTTCACCAACGACCAGATAGATAGCCTTGAGAAAGAGTTCGAGCGAACGCACTACCCGGACGTGTTCGCGCGGGAGCGGCTGGCCGAAAAGATTG

III-F

GATTGCCTGAGGCACGTATCCAGGTCTGGTTCTCCAACCGACGCGCGAAATGGCGACGGGAAGAGAAGTTGCGCAGCCAACGGAGAGACGCGCCCGCCTCGCCCCCCGCACCCCCCGCGCGCCTGCCGCTCAACGGAGGGTTCAACTCTATGTA

III-R

TAGCCCGATACCACAACCCATCGCCACCATGGGTGATACGTATAGCTCAATGTCTGGCGGGCTGTCGTCGTCGTGTCTCCAGCAGCGAGACAGCGGGTACCCTTACATGTTCGGCGACGTGCTCGGCAGCGGCGGCTACTCACGGGCGCCAGCTGCGCATCAACAGCACGCGGCATACTCACAACCACAAGCGGCAGGCAGTACTGGTGTGATATCGGCGGGCGTGAGCGTCCCTGTGCAAATACCTTCGCAGGGGCCGGACCTCGCGTCGAATTATTGGGGACGGCTTCAGTGATCTCAGCTATTCGGGTTCCCGCATCGACTGCTCTCGATGCCTGAGAACCCGACCACCGTGCCCGAGTTGCCATCTAGCGGCGACATAGCGCACAACTACGCCGACTCTCACTTGTCTATGAACGCTTTAAACATGACAGCCGGCCATGACAGCGCCGTTTTGGCGGGAGATATGCAAGAGAACTCTGTGTCGTAAACAATCGAAATCGAACACATATTGTGAAATAAAAAGTTAATACGCAATACCCAGTGGTTATTTTTTAGTGTTTTAATGGTGCAACGTATTTATTTGTGTATAGTTTTTGTATTAACGTGTTACGGCTAAGCAACTGATGATGTTCAAATGTAGGAGTAAGTAATGGTCGTACACTTTGAGCTATGTTGCGTTGCTATTCGACATACATACGTTCTTTTAACCTACTCATTTGCTTGCTAATAAACTAGTGTACATTTTGTGCAGCATGTTTCATTGTGTATGATCGAATAAATTATAGTGAACAATAGTGGCGTTATTGTAAATATATAGAGCGCCATCTATTGTCGAGTAGAGGAAGTCTCCTCCGCAATTCGTTTTCCTCGACATGAAATTAAATGTAAATTGTTTGTGCCAACGTTTGTAATAATTGAGTGTTCCACGCCCTATGCGAGTTCCAACCCTTTCATTTCTTTGATGTATGCAATGGGGGTGCAAAATGTTAATGCTGTAGAGAAAAACGTGGCAATAAGCTTCGTTACAACGATTTGTTTCTCACCCCCTGCATCGGGGCAAATTGTCACAGACACAGACGAAGCGTGTTTGTATATTTTGTATCATAAATATAAATCAATTGACATTTACCATACATAATTAGTATTTTTAGGGAAGAAAATTTTCAAAAAACAGGGCCTTCGATTCTTTTATACCTTGGATGCACTAGTTATCGAATACACTGTTCATTGGTAGATAATTAAAGTCCACTTACCTGGCATCAAATTATTTAAGACTCAAAGTAACATATGTTTTTTTAATGCTAAATTGGAAGCAATCAAAAAATTCTAGCTTTGAAAACAAGGTTTTTACCAAACTTTGCATTCAAAGCTACCTACAATTTCTGTTACCACACTTGGAACTATGTAAAAGCATGTAAATATTATTATTTACTAGCGAACGCTCGCGACTTCGTCCGCCCTTAGACCCAGATGTAAAAAA

(B) Sequencing results using the primers used in the study

>Ef_PD_HD_IF_IIIR

GANCTGNNGCACAGTGCGGCAATGGGNGGCGGCGCCCTCTTCGGGTGCTCCTCTGCCNNNNNCAGCGGGATCAACCAGCTGGGCGGGGTGTACGTCNNCNNNNNACCCTTGCCTGACTCCACGCGGCAGAAGATAGTGGAGCTGGCGCACTCGGGGGCGCGGCCGTGCGACATCAGCAGGATCCTGCAGGTGTCCAACGGCTGCGTGTCCAAGATACTAGGCAGGTACTACGAGACGGGGTCGATCAAGCCCCGTGCGATCGGCGGGTCAAAGCCGCGGGTCGCAACGACCCCGGTGGTTTCNAAGATCGCTGACTACAAGCGGGAGTGCCCTTCAATCTTCGCGTGGGAGATCCGGGATCGCCTGCTTANCGAAAACGTCTGCAATAACGACAATATCCCTAGCGTGTCATCAATCAACCGCGTGCTAAGGAACCTCGCATCACAGAAGGAGCAAGCAGCATCAGCGCAGAACGACAGCGTGTATGAGAAGCTGAGGATGTTCAATGGGCAGGCNNCNACNGGGTGGTGGTACCCTGGACTACCCGCCGCGCCTACCGCACCCGCGCTACCNGCACCACTACCCCCCCAGCTGAATAGGCCGACGTCCGAAGACCATAAACGAGATACCTTACAATCGGAGGCCGGGTCNNNANGGNNNCAGCNANNNCGCGTCNTCAGGAGACGAGGACTCGCANATGAGGCTGANGCTNANNAGGAAGCTGCNGAGNANCNNGANCNTCCTTCACCAACGANCNNGATANATAGNCTTGANAANNANNTCNAGCGAACCNCACTACCCGGANCGTGNTCG

>Ey_PD_I

AGNNGCCTTCTGNAAGATGCCGCACAAAGATGAGCTGATGCACAGTGCGGCAATGGGTGGCGGCGCCCTCTTCGGGTGCTCCTCTGCCGGCCACNGCGGGATCAACCAGCTGGGCGGGGTGTACGTCAACGGGAGACCCTTGCCTGACTCCACGCGGCAGAAGATAGTGGAGCTGGCGCACTCGGGGGCGCGGCCGTNCGNC

>Ey_PD_II

CGCCNGCTNAGCGANNCGTCTGCAATAACGACAATATCCCTAGCGTGTCATCNNTCAACCGCGTGCTAAGGAACCTAGCATCACAGAAGGAGCAAGCAGCATCAGCGCAGAACGACAGCGTGTATGAGAAGCTGAGGATGTTCAATGGGCAGGCNGCCACAGGGTGGTGGTACCCTGGACTACCCGCCGCGCCTACCGCACCCGCGCTACCAGCA

>Ey_HD_III

ACGTGTNCGCGCGGGAGCGNNTGGCCGAAAAGATTGGATTGCCTGAGGCACGTATCCAGGTCTGGTTCTCCAACCGACGCGCGAAATGGCGNCGGGAAGAGAAGTTNCGCAGCCAACGGAGAGACGCGCCCGNCTCGCCCCCCGCACCCCCCGCGCGCCTGNCGCTCAACGGNGG

(C) Alignment of the sequenced regions to the eyeless mRNA highlighting the paired domain and homeodomain

Ey_Complete CCGCCACCACGCTGGTGGGGGGGCTGGTGGCGCCGCCGCGGGAACCCACCACGGCGGCAG 60

Ef_PD_HD ------------------------------------------------------------

Ey_Complete AAGCCTTCTGGAAGATGCCGCACAAAGATGAGCTGATGCACAGTGCGGCAATGGGTGGCG 120

Ef_PD_HD -----------------------------GANCTGNNGCACAGTGCGGCAATGGGNGGCG 31

** *** ****************** ****

Ey_Complete GCGCCCTCTTCGGGTGCTCCTCTGCCGGCCACAGCGGGATCAACCAGCTGGGCGGGGTGT 180

Ef_PD_HD GCGCCCTCTTCGGGTGCTCCTCTGCCNNNNNCAGCGGGATCAACCAGCTGGGCGGGGTGT 91

************************** *****************************

Ey_Complete ACGTCAACGGGAGACCCTTGCCTGACTCCACGCGGCAGAAGATAGTGGAGCTGGCGCACT 240

Ef_PD_HD ACGTCNNCNNNNNACCCTTGCCTGACTCCACGCGGCAGAAGATAGTGGAGCTGGCGCACT 151

***** * ***********************************************

Ey_Complete CGGGGGCGCGGCCGTGCGACATCAGCAGGATCCTGCAGGTGTCCAACGGCTGCGTGTCCA 300

Ef_PD_HD CGGGGGCGCGGCCGTGCGACATCAGCAGGATCCTGCAGGTGTCCAACGGCTGCGTGTCCA 211

************************************************************

Ey_Complete AGATACTAGGCAGGTACTACGAGACGGGGTCAATCAAGCCCCGTGCGATCGGCGGGTCAA 360

Ef_PD_HD AGATACTAGGCAGGTACTACGAGACGGGGTCGATCAAGCCCCGTGCGATCGGCGGGTCAA 271

******************************* ****************************

Ey_Complete AGCCGCGGGTCGCAACGACCCCGGTGGTTTCGAAGATCGCTGACTACAAGCGGGAGTGCC 420

Ef_PD_HD AGCCGCGGGTCGCAACGACCCCGGTGGTTTCNAAGATCGCTGACTACAAGCGGGAGTGCC 331

******************************* ****************************

Ey_Complete CTTCAATCTTCGCGTGGGAGATCCGGGATCGCCTGCTTAGCGAAAACGTCTGCAATAACG 480

Ef_PD_HD CTTCAATCTTCGCGTGGGAGATCCGGGATCGCCTGCTTANCGAAAACGTCTGCAATAACG 391

*************************************** ********************

Ey_Complete ACAATATCCCTAGCGTGTCATCAATCAACCGCGTGCTAAGGAACCTAGCATCACAGAAGG 540

Ef_PD_HD ACAATATCCCTAGCGTGTCATCAATCAACCGCGTGCTAAGGAACCTCGCATCACAGAAGG 451

********************************************** *************

Ey_Complete AGCAAGCAGCATCAGCGCAGAACGACAGCGTGTATGAGAAGCTGAGGATGTTCAATGGAC 600

Ef_PD_HD AGCAAGCAGCATCAGCGCAGAACGACAGCGTGTATGAGAAGCTGAGGATGTTCAATGGGC 511

********************************************************** *

Ey_Complete AGGCAGCCACAGGGTGGTGGTACCCTGGACTACCCGCCGCGCCTACCGCACCCGCGCTAC 660

Ef_PD_HD AGGCNNCNACNGGGTGGTGGTACCCTGGACTACCCGCCGCGCCTACCGCACCCGCGCTAC 571

**** * ** *************************************************

Ey_Complete CAGCACCACTACCCCCCCAGCTGAATAGGCCGACGTCCGAAGACCATAAACGAGATACCT 720

Ef_PD_HD CNGCACCACTACCCCCCCAGCTGAATAGGCCGACGTCCGAAGACCATAAACGAGATACCT 631

* **********************************************************

Ey_Complete TACAATCGGAGGCCGGGTCGGA-CGGGAACAGCGAGCACGCGTCATCAGGAGACGAGGAC 779

Ef_PD_HD TACAATCGGAGGCCGGGTCNNNANGGNNNCAGCNANNNCGCGTCNTCAGGAGACGAGGAC 691

******************* ** **** * ****** ***************

Ey_Complete TCGCAGATGAGGCTGAGGCTGAAGAGGAAGCTGCAGAGGAACCGGACCT-CCTTCACCAA 838

Ef_PD_HD TCGCANATGAGGCTGANGCTNANNAGGAAGCTGCNGAGNANCNNGANCNTCCTTCACCAA 751

***** ********** *** * ********** *** * * ** * **********

Ey_Complete CGACCAG-ATAGATAGCCTTGAGAAAGAGTTCGAGCGAAC-GCACTACCCGGAC-GTGTT 895

Ef_PD_HD CGANCNNGATANATAGNCTTGANAANNANNTCNAGCGAACCNCACTACCCGGANCGTGNT 811

*** * *** **** ***** ** * ** ******* *********** *** *

Ey_Complete CGCGCGGGAGCGGCTGGCCGAAAAGATTGGATTGCCTGAGGCACGTATCCAGGTCTGGTT 955

Ef_PD_HD CG---------------------------------------------------------- 813

**

Ey_Complete CTCCAACCGACGCGCGAAATGGCGACGGGAAGAGAAGTTGCGCAGCCAACGGAGAGACGC 1015

Ef_PD_HD ------------------------------------------------------------

Ey_Complete GCCCGCCTCGCCCCCCGCACCCCCCGCGCGCCTGCCGCTCAACGGAGGGTTCAACTCTAT 1075

Ef_PD_HD ------------------------------------------------------------
